# Umbilical needling therapy for neck pain during pregnancy: A case report

**DOI:** 10.1097/MD.0000000000040989

**Published:** 2024-12-20

**Authors:** Lifang Zheng, Shana Yao, Yabei Jin, Zhanling Sun

**Affiliations:** a Acupuncture and Moxibustion Department, Zhejiang Hospital of Integrated Traditional Chinese and Western Medicine, Hangzhou, China; b Zhejiang Chinese Medical University, Hangzhou, China.

**Keywords:** case report, holographic umbilical theory, neck pain, pregnancy, umbilical needle

## Abstract

**Rationale:**

Neck pain during pregnancy is a common disease during pregnancy. It is urgent to find a safe and effective method without side effects. Acupuncture therapy including umbilical needling has been widely used in many diseases. But there is no report in such cases.

**Patient concerns:**

We report a case of umbilical needling therapy for neck pain during pregnancy and explore its possible mechanism of action. A 35-year-old Chinese woman was pregnant for more than 7 months and had neck pain for 1 week.

**Diagnoses:**

Neck pain was diagnosed in Western medicine, and neck arthralgia in traditional Chinese medicine. The syndrome differentiation was wind-cold arthralgia.

**Interventions:**

One patient with neck pain during pregnancy was treated with umbilical needling. According to the 12 branches and the 8 trigrams in the umbilicus, the umbilical needling Zhuque 3 acupuncture and di Tiantai guayi acupuncture were used to treat neck pain during pregnancy. The clinical improvement was evaluated by the changes in clinical symptoms and signs and the improvement rate of the Visual Analogue Scale score.

**Outcomes:**

After 7 times umbilical needling treatments, the improvement rate of neck pain symptoms and Visual Analogue Scale score of pregnant women reached 100%.

**Lessons:**

These results show that umbilical needling therapy can effectively improve the clinical symptoms of neck pain during pregnancy without affecting the fetus. Umbilical needling in neck pain treatment during pregnancy should be further investigated.

## 1. Introduction

Neck pain during pregnancy refers to neck pain that occurs during pregnancy, even accompanied by adverse activities which interfere with pregnant women’s work, daily activities, and sleep. Although recent studies have shown that acupuncture and moxibustion is a valuable and safe treatment for pregnancy-related neck pain, the use of acupuncture and moxibustion in the pregnancy is limited due to the fear that some “taboo points” of acupuncture and moxibustion will cause abortion or adverse effects on the fetus. Therefore, we use Qi’s umbilical needling therapy to select a single Shenque acupoint to treat neck pain during pregnancy from the perspective of simple operation, few and precise acupoints, significant curative effect, and safety.

## 2. Case report

A 35-year-old Chinese woman was pregnant for >7 months and had neck pain for 1 week. The patient complained that after waking up in the air-conditioned room, her neck muscles were stiff, adverse activities accompanied the lateral pain, and her neck could rotate about 30° to 1 side. She could not lie on her back or side at night. She could only sleep by the bedside with a U-shaped pillow and needed the help of her family to get up. She had been treated with Shangjin ointment and local acupuncture, but there was no significant improvement. The 4 diagnoses of traditional Chinese medicine show that the tongue is light red, the moss is white, and the pulse is smooth. Neck pain was diagnosed in Western medicine, and neck arthralgia in traditional Chinese medicine. The syndrome differentiation was wind-cold arthralgia.

## 3. Acupuncture treatment

**Figure 1.**
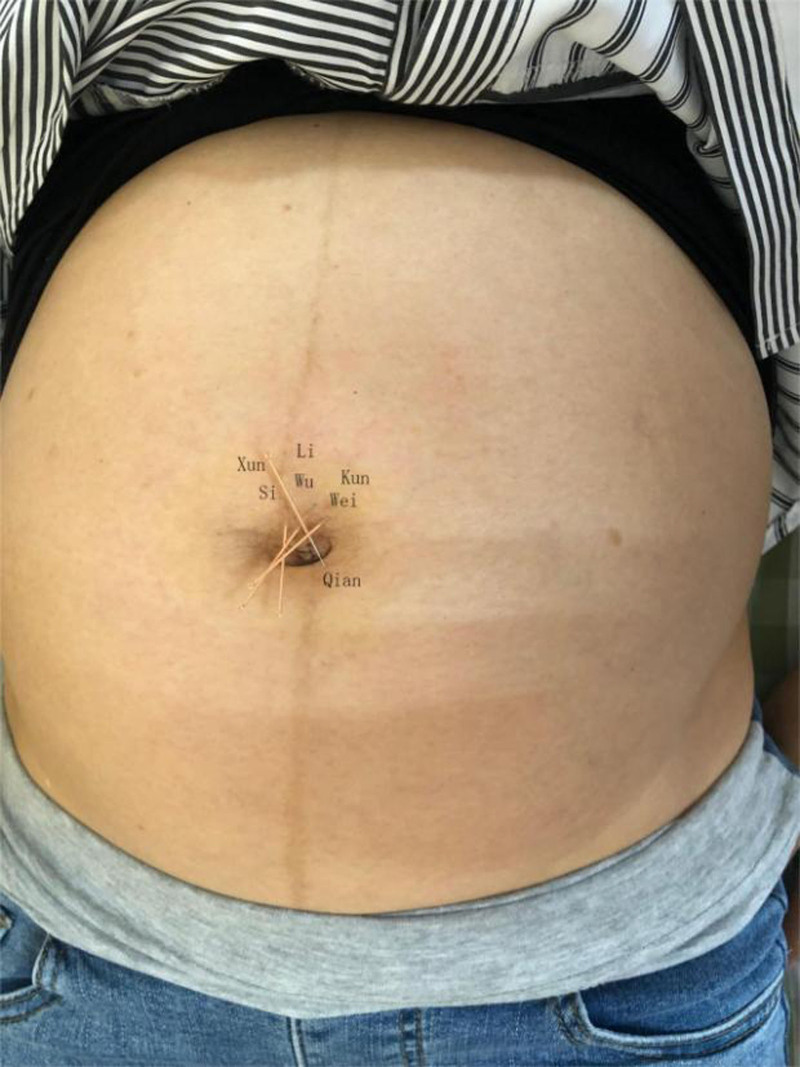
Picture of patient during treatment.

Umbilical needling treatment is based on dispelling wind and dispersing cold, warming and dredging meridians, and soothing the fetus. Select the navel needle prescription: Zhuque 3 needles (Si, Wu, and Wei) + Di Tiantai (Kun and Qian). The patient took the supine position and exposed the abdomen. After routine disinfection of Shenque point, 0.25 mm was selected × 25 mm Jiachen brand disposable sterile acupuncture needle, with the navel core as the center, twist the needle along with the upper 1/3 of the navel wall at the Si, Wu, Wei, and Qian positions (Fig. 1). The needle entry angle is 15° to the abdominal wall skin, and the acupuncture depth is 8 mm. After acupuncture, the needle handle is connected without lifting and inserting. The needle is kept for 30 minutes, once daily, for 7 consecutive days.

## 4. Clinical outcomes

The visual analogue scale (VAS) score before treatment was 6. During the first umbilical needling, the patient was instructed to move her neck appropriately, and the symptoms of neck pain have significantly improved compared to before. The cervical spine that cannot be moved can now be moved appropriately. But there was still pain at the Dazhui acupoint When the neck tilts back. The VAS score was 2. After the second umbilical needling treatment, the patient’s neck pain and restricted mobility have been largely relieved. The VAS score was 1. After 1 week of umbilical needling treatment, the neck pain disappeared. It was evaluated according to the changes in clinical symptoms and signs and the improvement rate of the VAS score. Improvement rate of VAS score = [(VAS score before treatment − VAS score after treatment) ÷ VAS score before treatment] × 100. The patient achieved clinical curative effect; the local pain symptoms of the patient’s neck spine completely disappeared, the neck spine function returned to normal, the VAS score was 0, and it had no effect on the fetus. After 1 month of follow-up, the patient’s neck spine activity was regular without obvious pain and discomfort, and she was satisfied with this treatment. The patient did not report any adverse events during the treatment and follow-up.

## 5. Discussion

In this case, umbilical needling therapy was effective for neck pain during pregnancy. Without apparent side effects, it improved the clinical symptoms and VAS neck pain score during pregnancy. Professor Qi Yong created umbilical needling therapy. It is an acupuncture therapy for acupuncture at Shenque’s point in the abdomen. It breaks the traditional Chinese medicine theory of banning acupuncture at Shenque Point.^[1]^ The acupoint selection method of the umbilicus needle is to enter the needle according to the orientation of the umbilicus Bagua hologram. The principle of manipulation is that there must be a direction for entering the needle, and the next needle must contain tonic and diarrhea.^[2]^ Guided by holographic umbilical theory, Yi Xue theory, umbilical traditional Chinese medicine theory, and umbilical time medicine theory, it is an acupuncture method to treat diseases by mobilizing human congenital meridians.^[3]^ Umbilical needling therapy is similar to other umbilical therapy methods. Stimulating around the umbilical cord can promote the regulation of human nerves and body fluids, improve immune function and adjust autonomic nerve function to prevent and treat disease.^[4]^ At the same time, umbilical needling is based on Yi medicine. It is believed that acupuncture at the umbilical part can mobilize the natural energy in the umbilical portion of the human body, cover the whole body from the habenula to the net through the innate meridian, impact the focus and regulate Yin and Yang.

**Figure 2.**
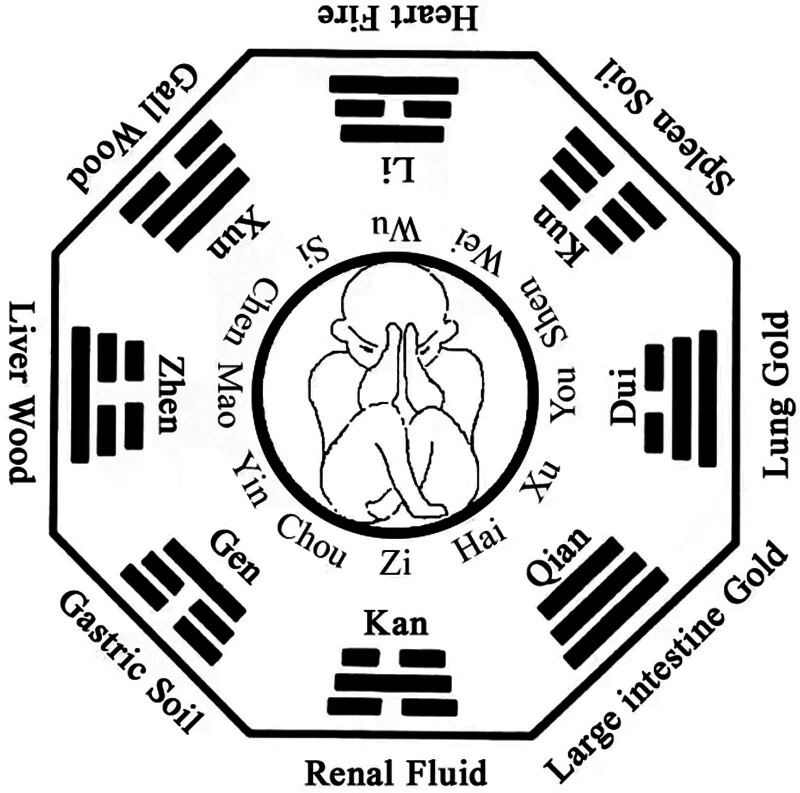
Holodiagram of umbilical Bagua.

Zhuque 3 needles is a kind of umbilical needling therapy. It selects 3 directions of Si, Wu, and Wei in the twelve Branches of the navel for acupuncture. The 3 directions of Si, Wu, and Wei correspond to Xun, Li, and Kun in the 8 trigrams hologram in the navel (Fig. 2). According to the 6 meridians syndrome differentiation theory, “the sun disease needs to be solved from Si to Wei,” Zhuque 3 needles correspond to “Mahuang Decoction” in Treatise on febrile diseases so it can treat some symptoms of Taiyang meridian caused by cold-evil entering the collaterals.^[5]^ The 3 directions of Si, Wu, and Wei correspond to the shoulder and neck spine in the umbilical Luoshu hologram. With the shooting method, the needle tip faces the direction of the disease to enhance the clinical treatment effect. The most significant difference between the treatment of pregnancy neck pain and ordinary neck spondylosis is that the woman is pregnant, and the fetus depends on the mother. Any treatment of the mother is bound to affect the fetus. Therefore, in treating pregnancy neck pain, we need to consider the fetal yuan and cooperate with the Qian position, which forms the earth heaven Tai divination acupuncture method with the Xun position of the Zhuque 3 needles. In the theory of traditional Chinese medicine, the 8 trigrams take the elephant Kun as the earth as Yin, the Qian as the sky as Yang, the Yin and Yang intersect, the upper and lower communicate, the heaven and the earth cross, and all things breed to form the “earth heaven Tai” divination, which means the intersection of heaven and earth and the elimination of all diseases, It can regulate Yin and Yang, stabilize the fetus and give consideration to fetal yuan. It can not only restrict the excess and deficiency of pregnancy neck pain but also won’t damage the fetus.

## 6. Conclusion and limitation

The investigation results of this case show that umbilical needling may be a safe, effective, and simple treatment option for neck pain during pregnancy. It needs further evaluation through future research to confirm its efficacy with a sufficient sample size and appropriate control group.

This study has the following limitations. This is a single case report and lacks a control group. The efficacy of acupuncture alone in treating this disease cannot be fully proved when combined with conventional treatment. Therefore, a larger sample size and additional controls are needed to support the efficacy of acupuncture. However, Umbilical needling is a good adjuvant treatment for pregnant patients.

## Author contributions

**Data curation:** Shana Yao.

**Formal analysis:** Zhanling Sun.

**Methodology:** Yabei Jin.

**Project administration:** Lifang Zheng.

**Writing – original draft:** Lifang Zheng.

**Writing – review & editing:** Zhanling Sun.
